# The effectiveness of air-free warming systems on perioperative hypothermia in total hip and knee arthroplasty: A systematic review and meta-analysis

**DOI:** 10.1097/MD.0000000000015630

**Published:** 2019-05-13

**Authors:** Shuyan Liu, Yu Pan, Qiancong Zhao, Wendy Feng, Hongyu Han, Zhenxiang Pan, Qianchuang Sun

**Affiliations:** a Department of Ophthalmology, The Second Hospital of Jilin University, Changchun, China; b Department of Genetics, The University of Alabama at Birmingham, Birmingham, AL; c Department of Anesthesiology and Resuscitology, Okayama University, Okayama, Japan; d Department of Cardiovascular Surgery, The Second Hospital of Jilin University, Changchun, China; e Department of Cell, Developmental and Integrative Biology, The University of Alabama at Birmingham, Birmingham, AL; f Department of Anesthesiology, The Second Hospital of Jilin University, Changchun, China.

**Keywords:** air-free warming, arthroplasty, forced-air warming, hypothermia, meta-analysis

## Abstract

**Background:**

Perioperative hypothermia is a common and serious complication during surgery. Different warming systems are used to prevent perioperative hypothermia. However, there have been no previous meta-analyses of the effectiveness of air-free warming systems on perioperative hypothermia in patients undergoing joint arthroplasty.

**Methods:**

We systematically searched PubMed, EMBASE, Cochrane Library, and China National Knowledge Infrastructure (CNKI) databases to collect randomized controlled trials (RCTs) from inception to August 2018. These RCTs compared the effects of air-free warming with forced-air (FA) warming system in patients undergoing joint arthroplasty. Postoperative temperature, core temperature during surgery, thermal comfort, blood loss and incidence of shivering and hypothermia were analyzed.

**Results:**

A total of 287 patients from 6 clinical studies were included in the analysis. In summary, there was no significant difference in the postoperative temperature (WMD −0.043, 95% CI −0.32 to 0.23, *P* = .758) between the air-free warming and FA warming groups. No statistical difference (WMD 0.058, 95% CI −0.10 to 0.22, *P* = .475) was found in core temperatures at 0 minutes during surgery between the air-free warming and FA warming groups. Furthermore, there was no statistical difference in thermal comfort, blood loss or incidence of shivering and hypothermia between the air-free warming and FA warming groups.

**Conclusions:**

Air-free warming system was as effective as FA warming system in patients undergoing joint arthroplasty.

## Abstract 2

Supplemental Digital Content is available in the text

## 1 Introduction

Hypothermia occurs frequently in patients undergoing joint arthroplasty,^[1–3]^ which can lead to a number of adverse outcomes including cardiac morbidity, surgical site infection, prolonged postanesthetic recovery, postoperative shivering, increased blood loss and longer hospital stays.^[4–7]^ Therefore, the use of active warming devices has become a standard procedure during surgery.

Forced-air (FA) warming is the most common method to prevent hypothermia in surgical patients.^[8]^ However, these convective warming systems have been shown to be potential sources of increased surgical site contamination due to the disruption of unidirectional laminar airflow.^[9]^ In addition, some studies have suggested that pathogenic organisms can be found in the hose of FA warming devices.^[10–13]^ By contrast, patient conductive warming devices have no noticeable impact on ceiling to floor ventilation in the operating room.^[9,14]^ Nevertheless, the debate about which warming device (convective warming or conductive warming) is superior in preventing perioperative hypothermia continues to be an area of argument. Therefore, we conducted a meta-analysis to provide evidence from RCTs to evaluate the effectiveness of air-free warming systems on perioperative hypothermia in patients undergoing joint arthroplasty.

## 2 Materials and methods

Studies were performed in accordance with the PRISMA protocol.^[15]^

### 2.1 Study search strategy

We systematically searched the PubMed, EMBASE, Cochrane Library, and China National Knowledge Infrastructure (CNKI) databases from inception to August 2018. Medical subject headings and text words “forced air warming, warming or warmer” and “arthroplasty or orthopedic” were used to search for trials of interest. Details of the search strategies are summarized in Supplementary Table S1. There were no language restrictions. In order to avoid omitting relevant clinical trials, we also searched conference summaries and reference for potential eligible reports.

### 2.2 Selection criteria

Inclusion criteria were as follows:

1. studies designed as randomized controlled trials (RCTs);
2. adult patients undergoing joint arthroplasty;
3. the test group treated with conductive warming, and the control group with FA warming;
4. outcomes such as postoperative temperature, core temperature during surgery, thermal comfort, blood loss and incidence of shivering and hypothermia.

Exclusion criteria were as follows:

1. non-RCTs;
2. reviews, letters, abstracts, editorials, or studies reporting insufficient data;
3. no control group.

### 2.3 Data extraction

Two reviewers (QCS, SYL) independently extracted data from the selected studies. The mean value and variance were for continuous variables while proportions were for dichotomous outcomes. If data were presented as sample size, median, range and/or interquartile range, the author of the trial was contacted to inquire if they could provide raw data. Failing that, we used some estimation formulas to estimate the mean and standard deviation.^[16]^ Extracted data included first author, publication year, country, group and method of warming, temperature device and site, type of anesthesia and outcomes. All these extracted data were summarized in Microsoft Excel and table format. The primary outcomes of this meta-analysis were postoperative temperature and core temperatures at 0, 30, 60, 90, and 120 minutes during surgery. The second outcomes were thermal comfort, blood loss, and incidence of shivering and hypothermia. Postoperative thermal comfort was evaluated with a visual analog scale (VAS) (0, extremely cold; 5, thermally neutral; and 10, extremely hot).

### 2.4 Assessment of quality and bias

To assure the quality of the eligible studies, risk of assessment was systematically and independently performed according to the Cochrane Collaboration's tool.^[17]^ The evaluation should include the following domains:

1. random sequence generation;
2. allocation concealment;
3. blinding of participants and personnel;
4. blinding of outcome assessment;
5. incomplete outcome data;
6. selective reporting;
7. other bias. Each of these domains was judged as low risk, high risk or unclear risk.

### 2.5 Statistical analysis

All statistical analyses were performed in Stata 14.0 (Stata Corp, College Station, TX) and Review Manager 5.3 (The Nordic Cochrane Centre, The Cochrane Collaboration, Copenhagen, 2014). Risk ratios (RRs) with 95% confidence intervals (CIs) were calculated for dichotomous data, and weighted mean differences (WMDs) with 95% CIs were calculated for continuous variables. Heterogeneity was measured by *I*^2^, with *I*^2^ > 50% indicating significant heterogeneity. If *I*^2^ < 50%, the fixed effects model was used; if *I*^2^ > 50%, a random effects model was used, and the heterogeneity was assessed. Subgroup analyses were performed for the outcome measures, according to warmed intravenous fluids (yes or no) and anesthesia (general anesthesia/spinal, spinal or general anesthesia). Sensitivity analyses were performed by excluding one study each time to evaluate the influence of a single study on the overall estimate.^[18]^ This is a meta-analysis. Thus, ethical approval was not necessary and the informed consent was not given.

## 3 Results

**Figure 1:**
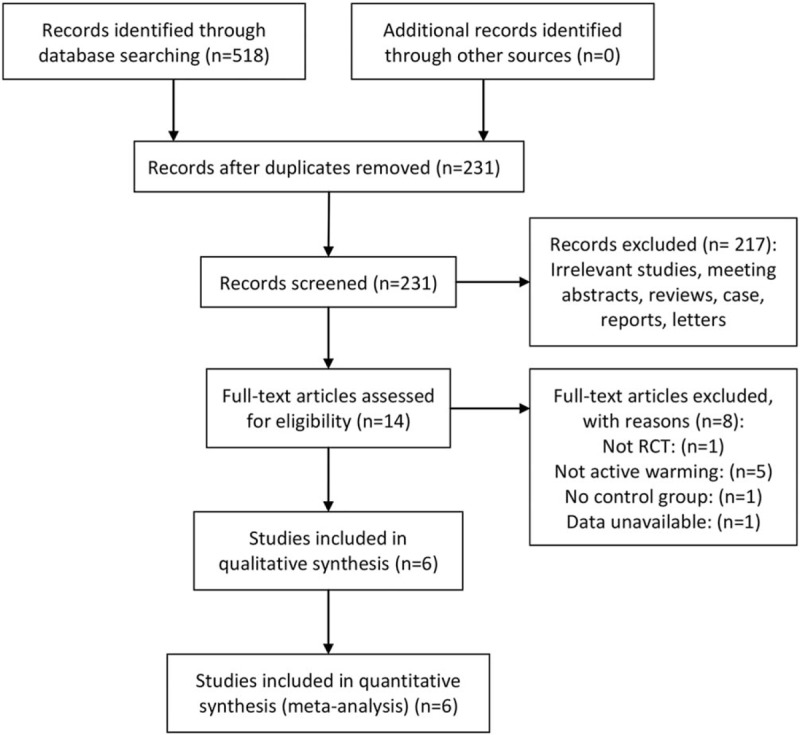
Flow chart of the review process.

**Figure 2:**
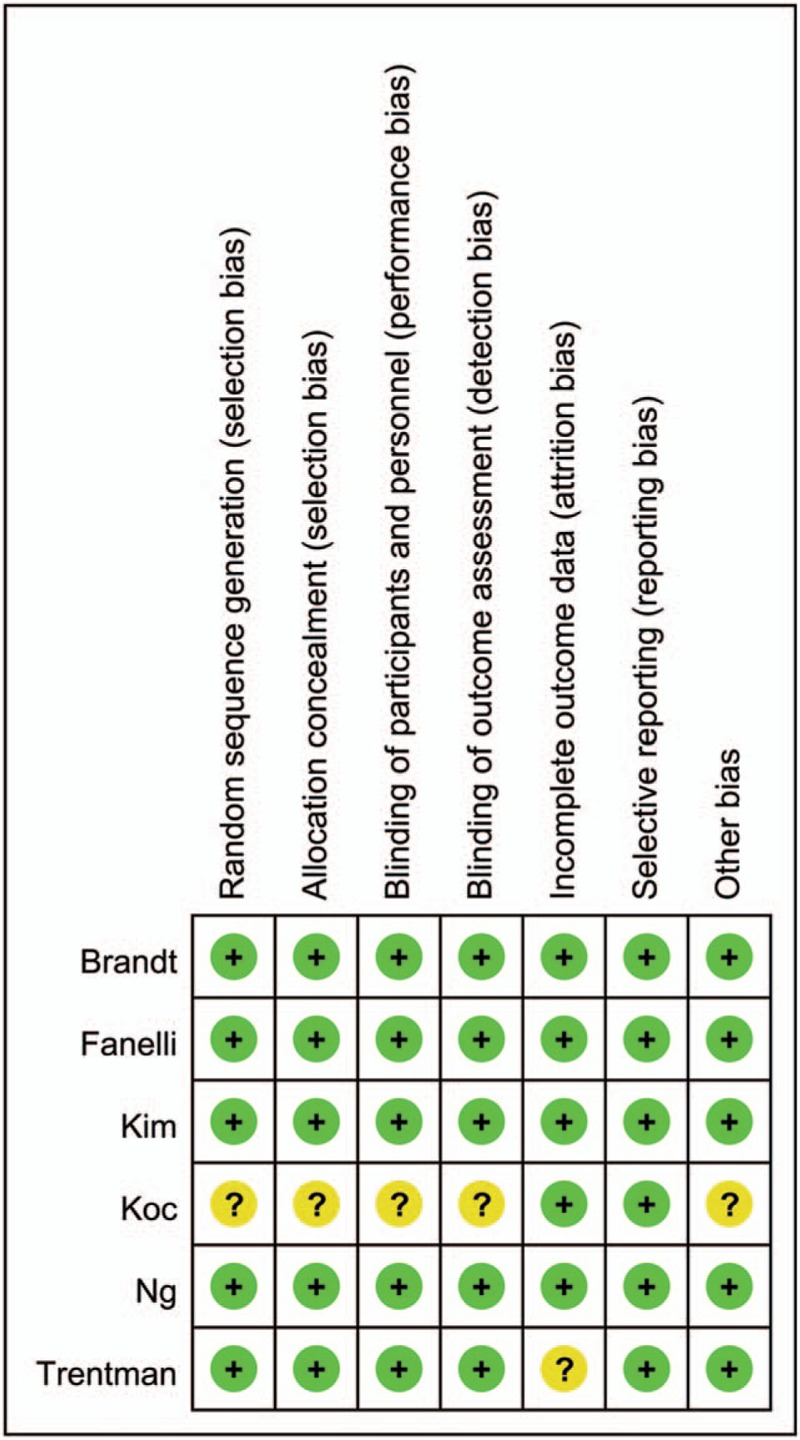
Risk of bias summary.

Figure 1 presents a summary of the study search process. A total of 518 relevant studies were initially identified. Of these, 231 were excluded due to duplication. After screening titles and abstracts, 217 more were further excluded. By reading the full text of the remaining 14 articles, nine were additionally excluded as they failed to meet inclusion criteria. Thus, six RCTs with 287 patients were ultimately assessed in this meta-analysis.^[3,19–23]^ The risk assessment of the included studies is presented in Figure 2. The majority of trials demonstrated a low risk of bias. Five of the reviewed studies clearly described the procedure of randomization, whereas the other one had minor deficiencies. Most of the trials were double-blinded for participants and outcome assessors.

Characteristics of the included studies are shown in Table 1. Three trials performed spinal anesthesia, and 1 trial performed general anesthesia (GA), and 1 trial received general/spinal anesthesia. Three studies received warmed intravenous fluids while the other three administered non-warmed intravenous fluids. Postoperative temperature was reported in all included trials. Meanwhile, only 5 studies monitored the core temperature continuously during surgery.

**Table 1:**
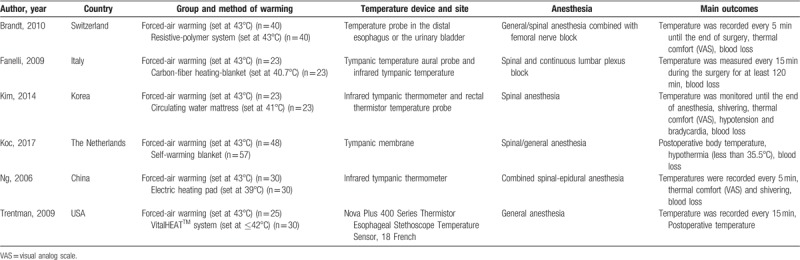
The general characteristic of the included studies.

**Figure 3:**
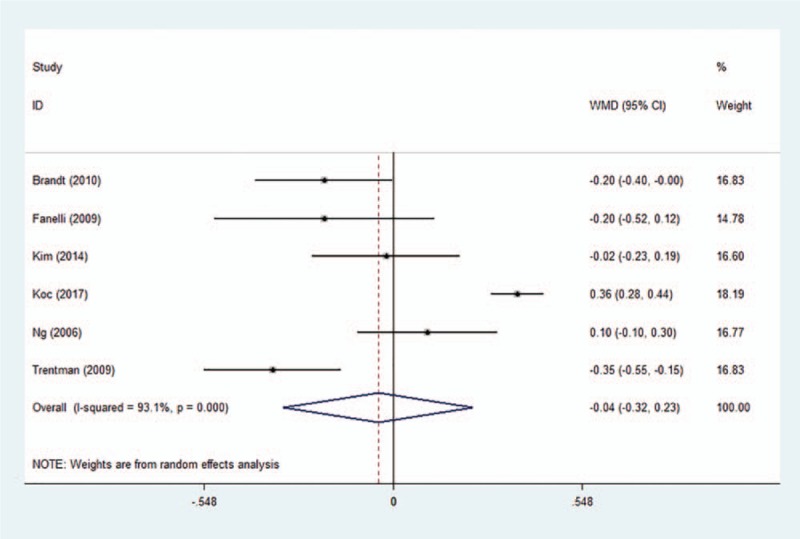
Forest plot of postoperative temperature between the two groups. WMD, weighted mean difference; CI, confidence interval.

**Figure 4:**
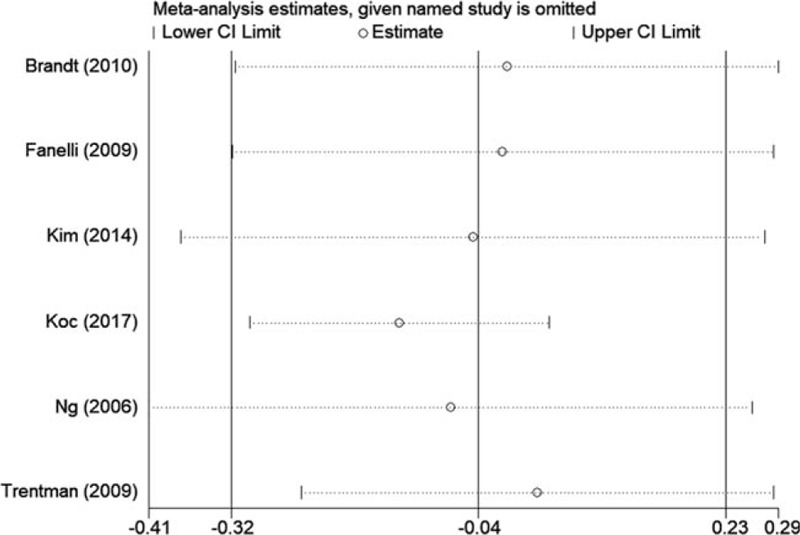
Sensitivity analysis of postoperative temperature between the two groups. CI, confidence interval.

The postoperative temperature was reported in all included studies. Pooled analysis demonstrated that air-free warming system was as efficient as FA warming system in patients undergoing joint arthroplasty (Fig. 3) (WMD −0.043, 95% CI −0.32 to 0.23, *P* = .758). Subgroup analyses are shown in Table 2. Warmed intravenous fluids (yes or no) and anesthesia (GA/spinal, spinal or GA) did not contribute to the heterogeneity. Sensitivity analysis did not significantly alter the summarized results (Fig. 4).

**Table 2:**
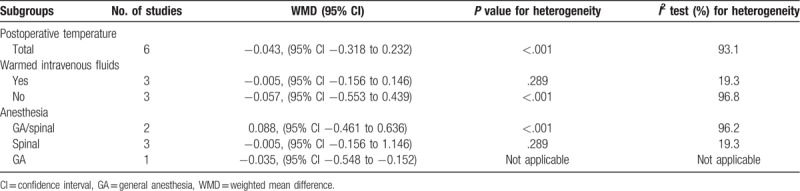
Subgroup analyses.

**Figure 5:**
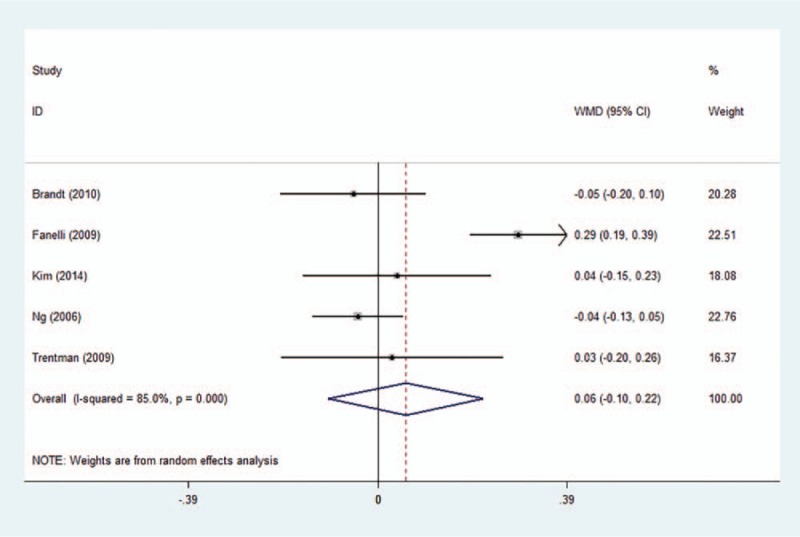
Forest plot of core temperature at 0 minutes during surgery between the 2 groups. WMD = weighted mean difference, CI = confidence interval.

The core temperatures at 0, 30, 60, 90, and 120 minutes during surgery were reported in 5 studies, which were summarized in Table 3. Pooled data revealed that the air-free warming system was as effective as the FA warming system at 0 minutes for maintaining body temperature (Fig. 5) (WMD 0.058, 95% CI −0.10 to 0.22, *P* = .475). Subgroup analyses of intravenous fluid and anesthesia types did not influence the pooled results (Supplementary Fig. S1, 2). Sensitivity analysis did not significantly alter the summarized results.

**Table 3:**
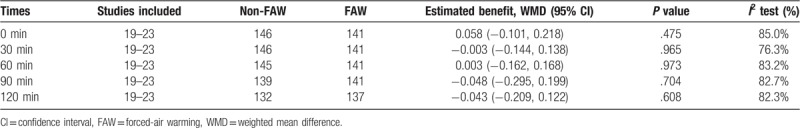
Core temperature at 5 different time points.

**Figure 6:**
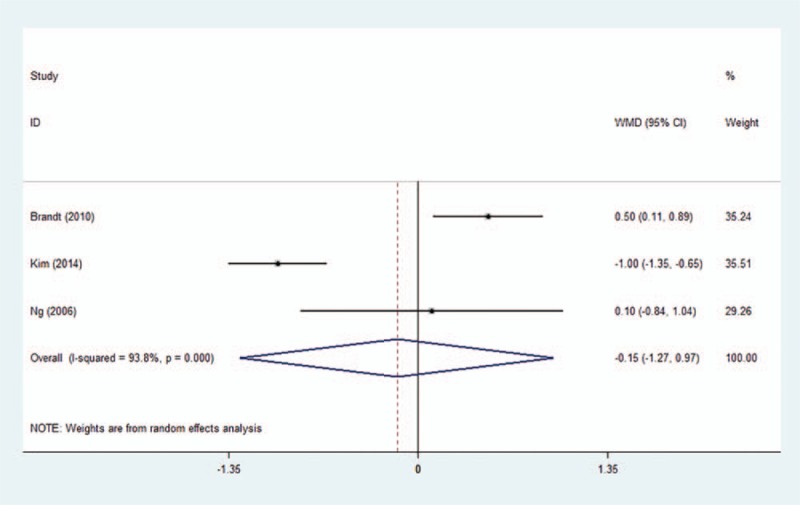
Forest plot of the postoperative thermal comfort between the two groups. WMD = weighted mean difference, CI = confidence interval.

Postoperative thermal comfort was provided in three trials. Pooled data indicated that patients’ thermal comfort was not different between air-free warming and FA warming groups(Fig. 6) (WMD −0.15, 95% CI −1.27 to 0.97, *P* = .793). Sensitivity analysis did not significantly alter the summarized results

**Figure 7:**
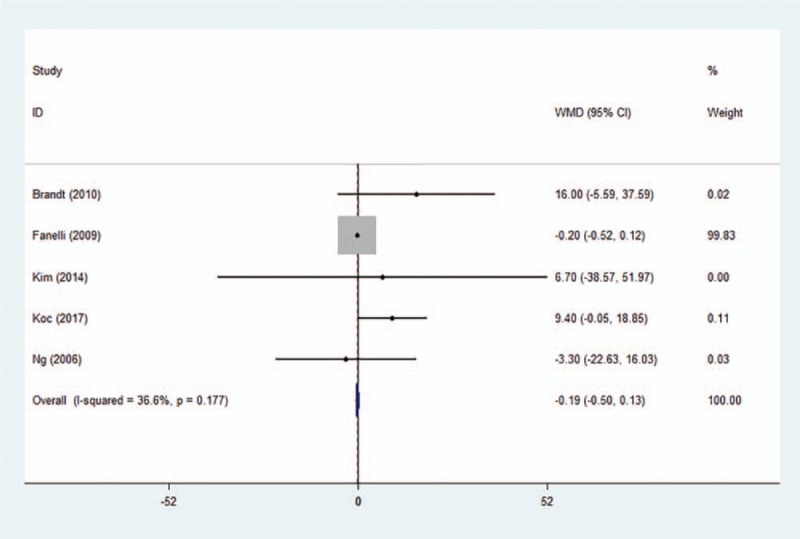
Forest plot of blood loss between the two groups. WMD = weighted mean difference, CI = confidence interval.

Five trials provided data about blood loss. Pooled analysis indicated that blood loss was not different between air-free warming and FA warming groups (Fig. 7) (WMD −0.19, 95% CI −0.51 to 0.13, *P* = .253).

For adverse events, pooled analysis showed no difference in the incidence of shivering and hypothermia between air-free warming and FA warming groups (Supplementary Fig. S3, 4).

## 4 Discussion

This is the first meta-analysis to evaluate the efficiency of air-free warming systems in patients undergoing joint arthroplasty. Our meta-analysis indicated that air-free warming systems were as efficient as FA warming systems in patients undergoing joint arthroplasty. No difference was found in the incidence of shivering and hypothermia between the air-free warming and FA warming groups.

Numerous factors contribute to the development of perioperative hypothermia including anesthesia-induced impairment of thermoregulatory control, long-term exposure to low temperatures in the operating room and altered distribution of body heat.^[24,25]^ Despite widespread recognition of adverse outcomes associated with hypothermia, maintaining normothermia in perioperative patient continues to present as a significant clinical problem. Therefore, almost every patient is dependent on active warming to prevent perioperative hypothermia, which is difficult to prevent with passive methods. Furthermore, some studies have shown that adequate prewarming before induction of anesthesia reduced the core-to-peripheral redistribution of body heat and produced higher core body temperatures during surgery.^[26–28]^

In this meta-analysis, no statistical difference was found in postoperative temperature between the air-free warming system and FA warming system in patients undergoing joint arthroplasty. Meanwhile, there was no significant difference in the core temperatures at 0, 30, 60, 90, and 120 minutes during surgery between the air-free warming and FA warming groups. In addition, this meta-analysis did not find any statistically significant difference in thermal comfort between the air-free warming and FA warming groups. Our results are consistent with another recent meta-analysis indicating that FA warming had similar effectiveness for preventing perioperative hypothermia compared with conductive warming devices (circulating-water garments, resistive heating blankets, and radiant warming).^[8]^

The efficacy and safety of FA warming for maintaining normothermia has been well documented.^[8,29]^ In this meta-analysis, no statistical difference was found in the blood loss or in the incidence of shivering and hypothermia between the air-free warming and FA warming groups. Nonetheless, the potential for laminar airflow disruption, which may be associated with surgical site infections, is present with the FA warming device.^[9–13,30]^ FA warming is commonly used during operation, since prewarming with FA warming can be a challenge due to lack of equipment and facility on the surgical ward.^[28]^ The non-forced air device does not impede the laminar airflow and reduce the risk of contamination of the surgical site. There are other benefits of non-air warming including less noise, less warming of the operating room (OR) environment and more thermal comfort for OR staff. Due to equal patient warming capabilities of FA warming and air-free warming devices, the application of devices with the least-associated risk should be fully explored.

This meta-analysis has several limitations worthy of consideration. First, six RCTs with 287 patients were included in this meta-analysis, so the sample size is too small to get an accurate result. Therefore, it is necessary to conduct a large-scale trial study to further investigate the performance of FW and air-free warming techniques. Second, there was significant heterogeneity for many of the study outcomes. In order to explore this heterogeneity, we performed subgroup analyses to account for different types of anesthesia and intravenous fluids. However, the subgroup analyses and sensitivity analyses did not appear to significantly alter the heterogeneity or statistical significance of results. Third, there were various different temperature measurement methods used and different sites of measurement, which may affect temperature readings. Fourth, there were also different temperature settings between studies, which might have an effect on the efficacy of warming. Fifth, we did not perform publication bias due to the limited number of included studies (less than 10 studies), thus publication bias may exist in this meta-analysis. Finally, our analysis was unable to draw conclusions regarding important outcomes such as surgical site infections and burn injuries.

In conclusion, based on the results from this meta-analysis, we demonstrated that air-free warming systems performed as efficiently as FA warming systems for maintaining normothermia in patients undergoing joint arthroplasty.

## Author contributions

**Conceptualization:** Qianchuang Sun, Zhenxiang Pan

**Data curation:** Qianchuang Sun, Shuyan Liu, Yu Pan, Qiancong Zhao, Wendy Feng, Hongyu Han.

**Formal analysis:** Qianchuang Sun, Shuyan Liu.

**Funding acquisition:** Zhenxiang Pan.

**Methodology:** Qianchuang Sun.

**Project administration:** Qianchuang Sun.

**Resources:** Qianchuang Sun.

**Software:** Qianchuang Sun, Shuyan Liu.

**Writing – original draft:** Shuyan Liu.

**Writing – review & editing:** Qianchuang Sun, Zhenxiang Pan.

## Supplementary Material

Supplemental Digital Content
