# Supplementary material for: The effectiveness of air-free warming systems on perioperative hypothermia in total hip and knee arthroplasty: A systematic review and meta-analysis
Source: Medicine (Baltimore). 2019 May 13;98(19):e15630. doi: 10.1097/MD.0000000000015630 (PMC6531108; doi:10.1097/MD.0000000000015630)

**Supplementary Table S2. Search strategies**

| **Search** | **Query** |
| --- | --- |
| #1 | Arthroplasty |
| #2 | Arthroplasty, Replacement, Hip |
| #3 | Arthroplasty, Replacement, Knee |
| #4 | Orthopedic |
| #5 | Orthopedic Surgery |
| #6 | Orthopedic Surgeries |
| #7 | Surgeries, Orthopedic |
| #8 | Surgery, Orthopedic |
| #9 | OR/#1-9 |
| #10 | forced-air warming |
| #11 | forced air warming |
| #12 | forced air warmer |
| #13 | warming |
| #14 | warmer |
| #15 | OR/#10-14 |
| #16 | #9 AND #15 |


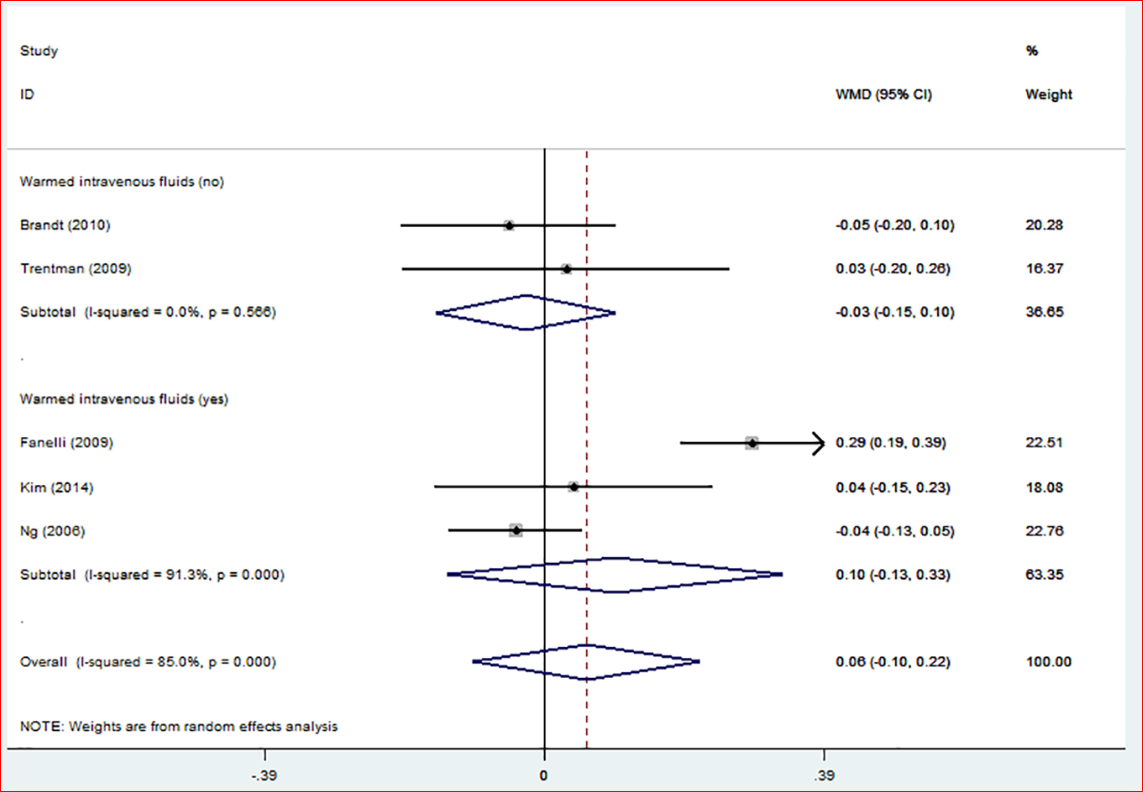


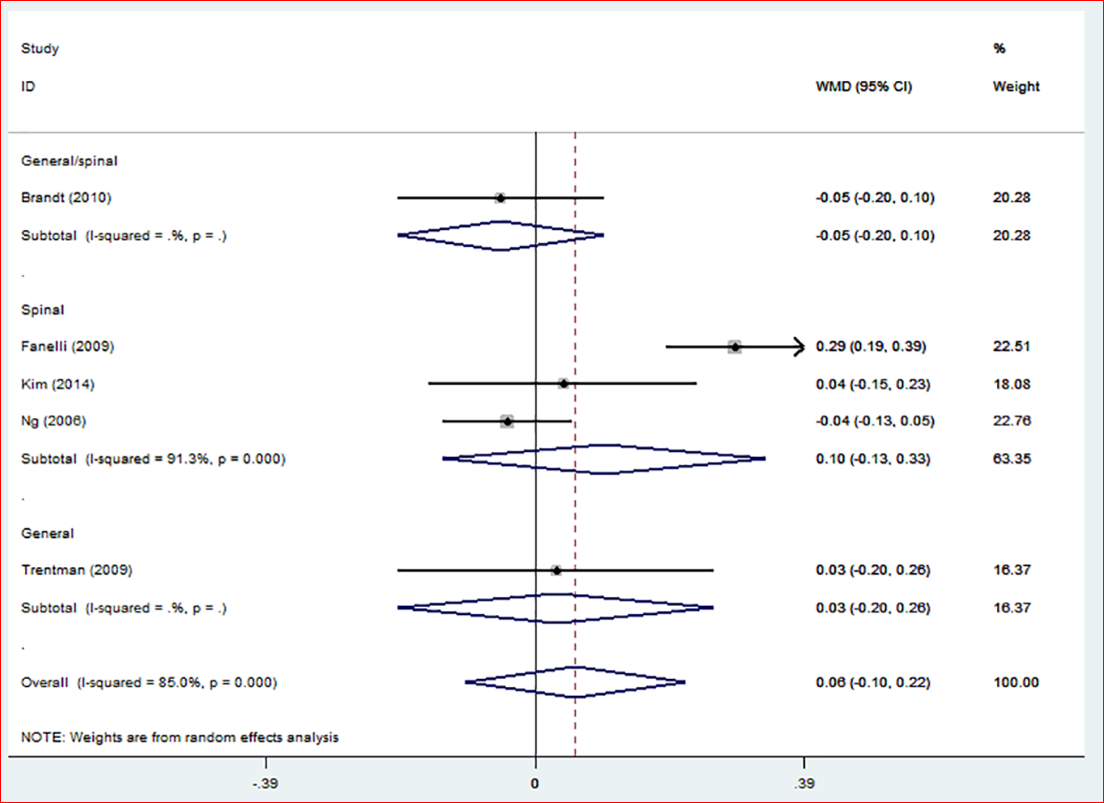


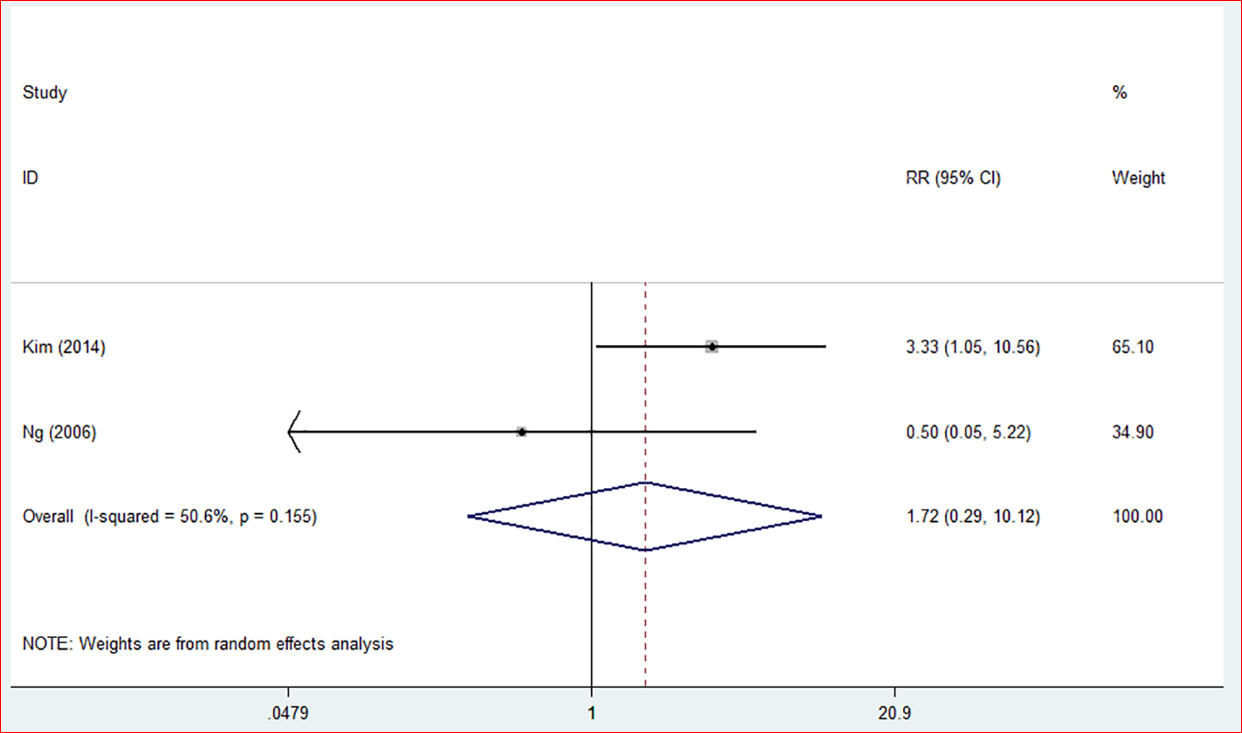


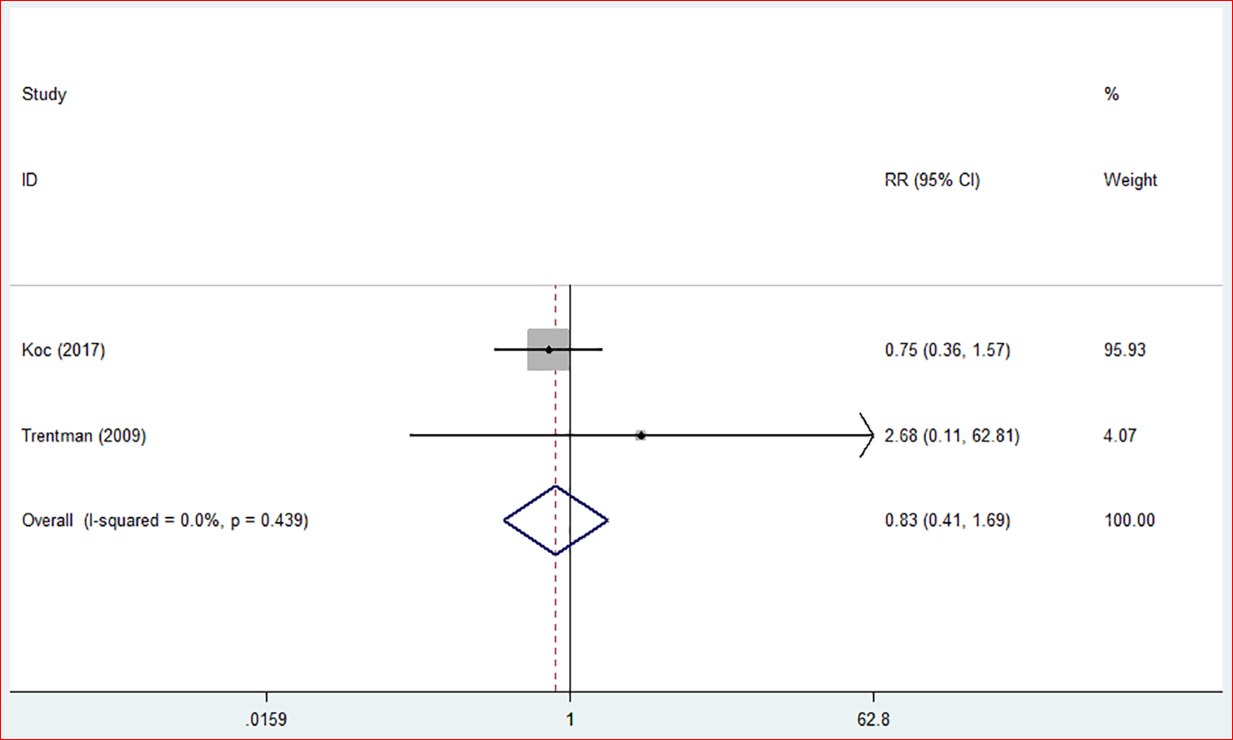

Supplement: Supplemental Digital Content [file medi-98-e15630-s001.doc]
